# The Law Affecting Medical Practitioners.—II

**Published:** 1902-11-22

**Authors:** J. E. R. Stephens

**Affiliations:** Barrister-at-Law, Author of "Digest of Public Health Cases"


### The Institutional Workshop.

### THE LAW AFFECTING MEDICAL PRACTITIONERS.—II.

By J. E. R. Stephens, Barrister-at-Law, Author of "Digest of Public Health Cases."

### Pretending to be Qualified.

To warrant a conviction for acting as or pretending to be a surgeon there must be unequivocal evidence that the party has so acted or pretended; it is not enough that he is so-called by persons whom he has attended professionally in the absence of evidence to show that he has done so on his own account and for his own profit (*Pedgrift* v. *Chevallier*, 8 W. R. 500).

H—— kept a shop where he dispensed medicines and gave advice; he had a diploma in the window in which he was described as "John Hamilton, Doctor of Medicine of the Metropolitan Medical College of New York," and he so held himself out to the world; and the magistrate reported that it was not satisfactorily proved that he was not entitled so to describe himself. He was not registered and held no qualifications which would entitle him to be registered under the Medical Act. The magistrate having dismissed a complaint brought against him under the Medical Act, 1858, s. 40, it was held that the decision of the magistrate was right, as the only evidence of any false pretence was that the person pretended to be what he really was, and also that it was a question of fact for the magistrate's decision rather than one of law for the Court. (*Carpenter* v. *Hamilton*, 37 L. T. 157).

A medical practitioner registered as Licentiate of the Society of Apothecaries, London, 1885, but not qualified to be registered as M.D., physician, or surgeon, under the Medical Act, 1858, placed "M.D." after his name on vaccination certificates, and in other ways described himself to the public as M.D., and as "physician" and "surgeon" in some cases with the further addition of "legally registered practitioner." It was held that he could be convicted of wilfully and falsely pretending to be a doctor of medicine, physician, and surgeon, contrary to section 40 of the Medical Act, 1858 (*Reg.* v. *Baker*, 56 J P. 406).

When the defendant signed a medical certificate as J.H., "Botanic surgeon, Boston, U.S." and had over his door, in large legible characters, his name, with the word "surgeon" after it, and in very small letters underneath, "Boston, U.S., not registered in England," and on a glass panel of his door his name, with the words "anti-registered surgeon" after it, the word "anti-registered" being in small letters and illegible except on close inspection, it was held that there was no evidence to convict him under the Medical Act, 1858, s. 40 (*Steele* v. *Hamilton*, 3 L.T. 322).

A druggist had attended a patient in the capacity of a medical man, and had sent in to him a bill for such attendances, headed, "Mr. P. to Thomas Andrews, M.D.", setting out a variety of charges for attendance and medicine. He subsequently wrote a letter, signed "Thomas Andrews, M.D." threatening legal proceedings unless the bill were paid, and he gave a receipt for the bill when paid, signing it in the same way. There was a coloured lamp over his shop door, on three sides of which the words and letters, "Thomas Andrews, M.D." were painted. He had obtained by the payment of a sum of money a diploma of doctor of medicine from the University of Philadelphia, in the United States, but he had never been in America, or studied, or passed any examination for such degree, and he was not registered under the Medical Act 1858. On appeal, from a conviction by justices under section 40 of that Act, for having unlawfully, wilfully, and falsely taken and used the name, title, description, and addition of M.D., and thereby implying that he was then registered under the Medical Act, whereas he was not so registered, it was held that the conviction was right and must be affirmed. (*Andrews* v. *Styrap*, 26 L. T. 704).

### Responsibility for Negligence.

Negligence, in which, though there is no criminal or dishonest conduct, but a gross want of that attention which the case of the patient requires, may be the subject of an action at law, and is also a serious misdemeanour and offence at common law, "because," as Blackstone observes, "it breaks the trust which the party had placed in his physician, and tends to the patient's destruction." Ignorance is also a great misdemeanour at common law, whether in a licensed or unlicensed practitioner, and at the same time the injury arising to any individual from his being a victim to this practice is a private wrong, for which he may recover adequate damages, but when a practitioner is duly qualified, of course there is considerable difficulty in fixing him with *crassa ignorantia*, however he may have erred in judgment. The circumstance of qualification affords a presumption in favour of the practitioner which juries will consider in such cases. There is no doubt that owing either to the death of the only person who could maintain the action, by the unskilful treatment, perhaps, of the medical practitioner, or the difficulty of producing the necessary evidence, which is obviously very great, or the hitherto uncertainty of medical practice, there has rarely occurred an opportunity of trying the point of the liability of the physician towards his patient for want of skill and attention, but the whole tenor of the books is that such an action can be supported. In *Seare* v. *Prentice* (8 East, 348–358) Lord Ellenborough, Chief Justice, said "that an ordinary degree of skill is necessary in a surgeon who undertakes to perform surgical operations, in the same manner as it is necessary for any other man to have it in the course of his employment, and I am ready to admit that a surgeon would be liable for *crassa ignorantia*, and would be justly responsible in damages for having rashly adventured upon the exercise of a profession, without the ordinary qualification of skill, to the injury of a patient." In *Slater* v. *Baker and Stapleton* (2 Wils. 359) an action against a surgeon and apothecary for ignorance and unskilfulness, tried before Chief Justice Wilmot, upon motion to set aside the verdict and damages which the plaintiff had obtained, the Court without even hearing the counsel for the plaintiff said: "He who acts rashly acts ignorantly; and although the defendants in general may be as skilful in their respective professions as any two gentlemen in England, yet the Court cannot help saying that in this particular case they have acted ignorantly and unskilfully, contrary to the known rule and usage of surgeons."

The liability of a medical man for negligence is not even terminated by the death of his patient, for by Lord Campbell's Act (9 & 10 Vict. c. 93), sections 1 and 2, whenever the death of a person is caused by some wrongful act, neglect, or default which would (if death had not ensued) have entitled the injured party to an action, then the person who would have been liable if death had not ensued shall be liable for damages, though the death was caused by an act amounting to felony; and the action shall lie for the benefit of the wife, husband, parent, and child of the deceased, and in the name of the executor or administrator, and the word "parents" is explained in the Act to include father, mother, grandfather, grandmother, stepfather, and stepmother, and the word "child" to include son, daughter, grandson, granddaughter, stepson, and stepdaughter.

The escape from liability is thus rendered almost impossible; and even the small weekly wages of a deceased son who contributed them towards the expenses of his parents' house, is evidence upon which the jury may act, though it is not shown that the sum contributed did more than cover the expense of the child's maintenance (*Duckworth* v. *Johnson*, 4 H. & N. 653). Proof must be given of a pecuniary loss through the death, sustained by the members of the family who sue, but the slightest pecuniary loss will support the action, or even a reasonable expectation of pecuniary advantage had the deceased lived, may be taken into consideration by the jury.

The mere fact that the treatment has not resulted in a cure is no proof of negligence, and the opinion of experts that it was unskilful is generally answered by that of other experts who testify favourably to the course pursued. If the person who attends the patient should be unqualified, he is, like the qualified man, liable for his conduct and equally bound to bring competent skill to the performance of the duty which he has undertaken, and is liable to an action for negligence if he fails to do so (*Ruddock* v. *Lowe*, 4 F. & F. 519). The only difference between his case and that of the qualified man consists in this, that a court or jury is much more likely to find that the treatment of which complaint is made was unskilful, and the plaintiff can therefore bring his action with a better chance of success.

From the nature of the cases to which their skill is directed, the medical profession are exposed to a special responsibility, from which the members of other professions are, as a rule, exempt. It is the legal duty of every person who undertakes to administer medical or surgical treatment, or to do any other lawful act of a dangerous character, requiring special knowledge, skill, attention, or caution, to employ in doing it a common amount of such knowledge, skill, attention, and caution. If it can be shown that a qualified, or unqualified, practitioner has caused the death of his patient through the lack of such knowledge, skill, attention, or caution, he may be convicted of manslaughter. But evidence of negligence which would be sufficient to be left to a jury in a civil action, is not necessarily sufficient to justify a conviction on a criminal charge. It should be shown that the accused acted recklessly, or with a criminal intent, or with such gross negligence as to come within the meaning of the word "felonious."

The law on the criminal responsibility of every man in such cases is thus laid down by Lord Hale:—"If a physician gives a patient a potion without any intent of doing him any bodily hurt, but with intent to cure or prevent a disease, and contrary to the expectation of the physician, it kills him, this is no homicide; and the like of a surgeon. And I hold their opinion to be erroneous that think, if it be no licensed surgeon or physician that occasions this mischance, then it is a felony, for physic and salves were before licensed physicians and surgeons, and, therefore, if they be not licensed according to the statutes, they are subject to the penalties in the statutes, but God forbid that any mischance of this kind should make any person not licensed guilty of murder and manslaughter." (1 Hale's Pleas of the Crown, 429.) And upon this latter point Sir William Blackstone appears to agree with Lord Hale. The correctness of Lord Hale's opinion has been recognised in many cases since. Thus in *Reg.* v. *Van Butchell* (3 C. & P. 632), Baron Hullock said that it made no difference whether the party was a regular or an irregular surgeon, adding that in remote parts of the country many persons would be left to die, if irregular surgeons were not allowed to practise. There is no doubt that there may be cases where both regular and irregular surgeons may be liable to an indictment, as there may be cases where from the manner of the operation, even malice might be inferred. Where a person who, though not educated as a surgeon, had been in the habit of acting as a man-midwife, and had unskilfully treated a woman in childbirth, in consequence of which she died, was indicted for murder, Lord Ellenborough said that to substantiate the charge, the prisoner must have been guilty of criminal misconduct, arising either from the grossest ignorance, or the most criminal inattention. One or other of these was necessary to make out a case of manslaughter (*Reg.* v. *Williamson*, 3 C. & P. 635). But where a person, grossly ignorant, undertook to deliver a woman and killed the child in the course of the delivery, it was resolved by the judges that he was rightly convicted of manslaughter (*Reg.* v. *Senior*, 1 M. C.C. 346).

The rule with regard to the degree of misconduct, which will render a person practising medicine criminally responsible is thus laid down by Mr. Justice Bayley in *Reg.* v. *Long* (4 C. & P. 398): "It matters not whether a man has received a medical education or not. The thing to look at is, whether, in reference to the remedy he has used, and the conduct he has displayed, he has acted with a due degree of caution, or on the contrary, he has acted with gross and improper rashness and want of caution. I have no hesitation in saying that if a man be guilty of gross negligence in attending to his patient, after he has applied a remedy or of gross rashness in the application of it, and death ensues in consequence, he will be liable to conviction for manslaughter."

